# Thermogenic Fat as a New Obesity Management Tool: From Pharmaceutical Reagents to Cell Therapies

**DOI:** 10.3390/biomedicines12071474

**Published:** 2024-07-04

**Authors:** Ying Cheng, Shiqing Liang, Shuhan Zhang, Xiaoyan Hui

**Affiliations:** 1 Zhongshan Hospital (Xiamen), Fudan University, Xiamen 361015, China; cheng.ying@zsxmhospital.com; 2 School of Biomedical Sciences, The Chinese University of Hong Kong, Hong Kong 999077, China; liangshiqingll@outlook.com (S.L.); 1155160276@link.cuhk.edu.hk (S.Z.)

**Keywords:** brown adipose tissue, beige adipocyte, β3 adrenergic receptor, brown adipose tissue transplantation, human induced pluripotent stem cell, obesity

## Abstract

Obesity is a complex medical condition caused by a positive imbalance between calorie intake and calorie consumption. Brown adipose tissue (BAT), along with the newly discovered “brown-like” adipocytes (called beige cells), functions as a promising therapeutic tool to ameliorate obesity and metabolic disorders by burning out extra nutrients in the form of heat. Many studies in animal models and humans have proved the feasibility of this concept. In this review, we aim to summarize the endeavors over the last decade to achieve a higher number/activity of these heat-generating adipocytes. In particular, pharmacological compounds, especially agonists to the β3 adrenergic receptor (β3-AR), are reviewed in terms of their feasibility and efficacy in elevating BAT function and improving metabolic parameters in human subjects. Alternatively, allograft transplantation of BAT and the transplantation of functional brown or beige adipocytes from mesenchymal stromal cells or human induced pluripotent stem cells (hiPSCs) make it possible to increase the number of these beneficial adipocytes in patients. However, practical and ethical issues still need to be considered before the therapy can eventually be applied in the clinical setting. This review provides insights and guidance on brown- and beige-cell-based strategies for the management of obesity and its associated metabolic comorbidities.

## 1. Introduction

Obesity has reached epidemic levels worldwide and has approximately tripled since 1975 [1]. It is estimated that nearly 40% of adults are overweight or obese, and this incidence will increase in the coming decades [1]. In addition, the number of children with obesity is steeply increasing, along with the fact that children of overweight and obese people have a higher chance of remaining obese into adulthood and developing non-communicable chronic diseases at a younger age, including diabetes and cardiovascular diseases [2].

Obesity is fundamentally caused by a positive energy balance, consequently leading to excessive lipid storage in the adipose tissue [1]. Under obese conditions, the functions of multiple organs are impaired, which results in disturbed whole-body energy homeostasis and thus gives rise to a vicious cycle forming the relapsing character of the disease. Therefore, obesity substantially increases the risk of numerous diseases, such as type 2 diabetes mellitus (T2DM), hypertension, cardiovascular diseases, metabolic-associated fatty liver disease (MAFLD), and certain types of cancer, all of which contribute to a decline in life quality and expectancy [3,4].

Due to the multiple means of biological adaptations (e.g., hormones and neurochemical defense adaptations), diet and exercise alone are often inefficient in maintaining long-term weight loss [5]. Luckily, brown adipose tissue (BAT) and “brown-like” beige adipocytes, which dissipate extra energy in the form of heat, have recently been detected in adult humans. The activation of these thermogenic fats has been suggested as an alternative strategy for treating obesity, diabetes, and cardiovascular diseases, which has been comprehensively reviewed elsewhere [6]. This review summarizes and discusses the current progress in applying thermogenic fat for therapeutic purposes, including the in vitro differentiation/mobilization of the cells and transplantation therapy.

## 2. Brown/Beige Adipose Tissue Biology

Adipose tissue constitutes approximately 20–25% of the total body weight in healthy subjects and is classified into three distinct types. White adipose tissue (WAT), which predominates in the body, serves as the energy storage reservoir as well as a thermal insulator and a cushion for internal organs. WAT is also an abundant source of hormones and bioactive factors [7]. White adipocytes are characterized by the presence of a single lipid droplet (unilocular) that pushes the nucleus and other organelles aside. WAT accumulates in individuals who are overweight and obese, especially in the abdominal region, and the amount of WAT is closely correlated with insulin resistance, diabetes, and other metabolic diseases [8]. In particular, the location of WAT is also essential for metabolic health. Epidemiological studies have shown that WAT in the visceral cavity is more harmful when it is deposited in the lower trunk of the body.

In contrast to WAT, BAT can dissipate energy through non-shivering thermogenesis [9]. Compared to white adipocytes, brown adipocytes are smaller in size (15–50 µm in diameter). Multiple lipid droplets (multilocular) and abundant mitochondria exist in each brown adipocyte, and the iron-rich mitochondria give them a brownish color. In addition to the morphological differences, brown adipocytes express a unique mitochondrial resident protein called uncoupling protein-1 (UCP1). As a proton leakage channel, UCP1 uncouples oxidative phosphorylation from ATP production, thus resulting in energy waste and heat generation [9].

Interestingly, unlike the classical BAT found in babies, many of these glucose-responsive adipocytes, which are called beige or brite (brown-in-white) cells, are inducible and convertible within adult human WAT [7,10]. Upon specific stimuli (such as cold acclimation), these otherwise quiescent beige cells are remodeled to demonstrate brown-like characteristics [10,11,12]. Therefore, beige adipocytes possess the properties of both white and brown adipocytes, acting as an energy reservoir in the quiescent condition while dissipating energy upon activation.

Both classical brown adipocytes and beige adipocytes have multilocular lipid droplets and a high mitochondria content. They are positive for UCP1 expression, giving them the general term of thermogenic adipocytes. However, beige adipocytes are not simply brown adipocytes in WAT [10,13]. Despite their similar features, classical brown and beige adipocytes are developmentally derived from distinct precursors and mobilized by both overlapping and different mechanisms [7]. Specifically, brown adipocytes originate from a *Myf5^+^* myogenic precursor, a special progenitor for skeletal muscle [13,14]. In contrast, beige adipocytes lack the historical expression of *Myf5^+^*. This suggests that brown adipocytes behave in more myocytic ways. Additionally, the UCP1 of beige adipocytes is only induced after appropriate stimuli; otherwise, the expression level is as low as that in white adipocytes [10,15]. Finally, they differ in other gene expression patterns despite both *UCP1* and other thermogenic genes being exhibited. Specific markers, such as *TMEM26*, *TBX1*, and *CD137*, are unique to beige adipocytes. On the other hand, brown adipocytes preferentially express *ZIC1* and *LHX8* [16]. Adipose tissue reservoirs in the supraclavicular and paraspinal regions do not consist of “pure” classical brown adipocytes. Instead, the brown-to-beige ratio is elevated when moving deeper. Despite the distinct developmental lineages and mechanisms of activation, whether brown and beige adipocytes have different functions remains unknown. Wu et al. suggested that fully stimulated brown and beige adipocytes contain comparable levels of UCP1 [10]. However, it will be interesting to investigate and compare the functions of these two adipocytes, such as their lipid-lowering, glucose uptake, anti-inflammation, and cytokine secretion patterns.

UCP1 plays a key role in adaptive thermogenesis through uncoupling the mitochondrial respiratory chain from ATP production. During a cold challenge, highly innervated sympathetic neurons are evoked and release catecholamine, mainly norepinephrine (NE). Followed by the provocation of β3-AR after NE binding, adenylyl cyclase (AC) is activated and converts ATP to cyclic adenosine monophosphate (cAMP). The downstream protein kinase A (PKA) is evoked to phosphorylate triacylglycerol lipase, resulting in lipid catabolism. Afterward, a large amount of free fatty acid is liberated and acts as a fuel to induce UCP1 expression. UCP1 uncouples the mitochondrial respiratory chain, speeding up the substrate oxidation rate to produce heat and lower ATP generation in brown fat [9].

However, UCP1 KO animal models still maintain cold resistance, indicating the presence of UCP1-independent thermogenesis. In beige adipocytes, specifically in UCP1 KO mice, pathways related to Ca^2+^ cycling were upregulated upon cold exposure, including sarco/endoplasmic reticulum Ca^2+^-ATPase2b (SERCA2b) [17]. This Ca^2+^ cycling uncoupled ATP production and resulted in heat production [17]. Furthermore, in thermogenic adipocytes, the creatine phosphorylation and de-phosphorylation cycle stimulates mitochondrial respiration, a process that occurs independently of UCP1 [18]. Josef et al. identified a futile triglyceride/fatty acid cycle as a novel mechanism in UCP1-independent thermogenesis [19]. This cycle involves concurrent triglyceride lipolysis and fatty acid re-esterification within the lipid droplets of brown adipocytes, thus accelerating ATP consumption and heat generation through the interaction with the mitochondrial electron transport chain (ETC) [19].

BAT was previously thought to be present only in human newborns. However, in the past decade, [18F]-fluorodeoxyglucose positron emission tomography/computed tomography (18^F^-PET/CT) unequivocally revealed the presence of functional BAT that extends from the anterior neck to the thorax in healthy adults [20,21]. The amount of BAT was inversely associated with age and BMI in either sex, although a higher mass and activity of BAT were detected in women compared to men [20]. Furthermore, a large-scale retrospective study recently revealed that the amount of BAT was closely correlated with cardiometabolic health [22]. There is also evidence that activation of BAT antagonized multiple types of cancer progression in preclinical animal models and in one lymphoma patient [23].

**Figure 1:**
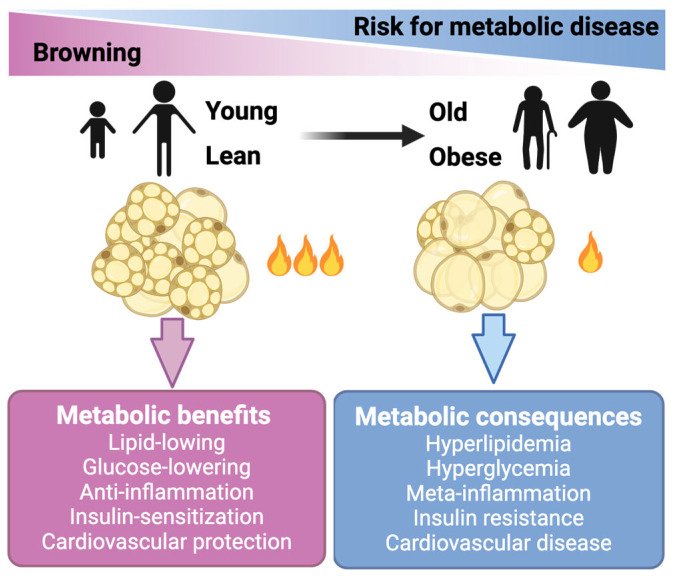
Brown and beige fat cells hold therapeutic potential for treating obesity and metabolic diseases. Brown and beige fat cells (adipocytes) generate heat to burn off extra amounts of glucose and lipids. Activation of these thermogenic adipocytes also confers benefits beyond lowering glucose and lipids. The activity of brown and beige adipose declines in obese and aging people.

The basal and maximum respiration of endogenous mature brown adipocytes were approximately 5–10 times higher compared to the stromal vascular fraction (SVF) cells or mature white adipocytes [24]. Therefore, these thermogenic adipocytes (beige and brown adipocytes) are active combustors of glucose and lipid. It is noteworthy that brown and beige cells also conferred a series of metabolic benefits beyond the direct glucose- and lipid-lowering effects, including anti-inflammation, insulin-sensitization, and anti-atherosclerosis (Figure 1) [25,26,27]. In line with these mechanistic studies, transplantation studies have demonstrated the essential role of BAT in improving adiposity, glucose metabolism, and insulin resistance in mice (reviewed in detail below). In clinical studies, the activation of brown/beige adipocytes by cold stimulation ameliorated glucose metabolism and insulin sensitivity in both healthy subjects and patients with T2DM (Figure 1) [28,29,30]. This evidence collectively highlights the therapeutic potential of thermogenic adipocytes in treating obesity and its related comorbidities.

## 3. Pharmaceutical Approaches for Activation of Brown and Beige Adipocytes

Recent research has provided insights into small molecules with the potential to activate BAT or beige adipocytes. Dietary components, including capsaicin, resveratrol, curcumin, green tea, menthol, and fish-derived omega-3 fatty acids, have shown signs of stimulating BAT and beige adipocyte activities, which at least partially explains their anti-obesity and anti-metabolic syndrome benefits. This information has been well summarized [31,32,33]. Therefore, the current review will focus on compounds beyond these categories that include either FDA-approved drugs or pharmaceutical reagents, for their effects on enhancing the thermogenic activity and energy expenditure rate in adipose tissues.

### 3.1. FDA-Approved Drugs Repurposed for Metabolic Diseases

#### 3.1.1. β3 Adrenergic Receptor (β3-AR) Agonist

β3 adrenergic receptor (β3-AR) is expressed in rodent brown adipocytes. It plays a vital role in engaging lipolysis and thermogenesis [34]. In humans with type 2 diabetes, short-term cold exposure activates brown/beige fat and improves insulin sensitivity [30]. However, cold exposure is clinically impractical owing to the inconvenience of the regimen, the discomfort, and the complex and indirect pathways elicited by the cold. Cold mimetics, i.e., nonspecific sympathomimetic drugs, also pose cardiovascular risks because of the widespread expression of sympathetic receptors, which limits their clinical application [35]. By this logic, β3-AR is a superior target since its expression is restricted in adipocytes and the urinary bladder. Although it is still under debate as to whether β3-AR is the predominant isoform expressed in human BAT [36], extensive efforts have been made to establish β3-AR agonism as a promising treatment regimen for metabolic disorders. Mirabegron is a selective β3-AR agonist with satisfactory bioavailability [37,38]. Sold under the brand name Myrbetriq^®^, it was initially approved by the U.S. Food and Drug Administration in 2012 for the treatment of overactive bladder (OBA) at daily doses of 25 or 50 mg. In recent years, a number of clinical trials have been carried out to explore the repurposing of this drug for treating obesity and metabolic diseases at a super-therapeutic dosage [11]. In particular, one study that explored an effective and safe dosage for individual use revealed that a 100 mg daily intake increased the skin temperature and energy expenditure in participants without any cardiovascular side effects, while increased blood pressure and heart rate were observed at 150 and 200 mg doses, respectively [39]. However, the exact β-AR isoforms that predominate in human brown and beige adipocytes are under debate [36,40]. Different β-AR isoforms may function in distinct brown and beige subtypes. Adipose-selective or even subtype-specific delivery of agonists might be of interest in future studies.

#### 3.1.2. Glucagon-like peptide-1 Receptor Agonists (GLP-1RA)

The glucagon-like peptide-1 receptor agonists (GLP-1RAs), including liraglutide and semaglutide, were initially used to manage T2DM due to their ability to amplify glucose-dependent insulin secretion [41,42]. They decrease plasma glucose concentrations, induce weight loss, have cardioprotective effects, improve insulin sensitivity, and reduce low-grade inflammation in humans [43]. In particular, a remarkable weight loss effect was observed clinically [44,45,46]. GLP-1RA acts directly on the brain and gastrointestinal tract to suppress appetite and delay gastric emptying. Whether GLP-1R is present in adipose tissue is less clear. It has been reported that liraglutide stimulates BAT thermogenesis and browning via hypothalamic AMP-activated protein kinase (AMPK) [47]. Liraglutide demonstrated a therapeutic effect on mitochondrial dysfunction in human adipocytes in vitro, promoting mitochondrial respiration and biogenesis [48]. Liraglutide also suppressed obesity and induced a brown-fat-like phenotype via the soluble-guanylyl-cyclase-mediated pathway in vivo and in vitro [49]. A recent study found that liraglutide promoted brown remodeling of visceral WAT by regulating miRNAs [50]. More importantly, in a longitudinal study of 25 patients with obese T2DM who were treated with exenatide or liraglutide for 1 year, both of these two GLP-1RAs elevated the energy expenditure rates of the subjects [47]. These observations suggest that long-acting GLP-1RAs control body weight at least partially through increasing energy consumption, in addition to their effect on regulating food intake. Repurposing the GLP-1RAs for obesity is quite popular in clinical scenarios. However, there is an upper limitation on weight loss in obesity, even without considering relapse after treatment cessation. Considerably obese patients also show resistance or poor response to the drugs. Furthermore, there are contraindications, such as pancreatitis and thyroid medulla carcinoma.

#### 3.1.3. Thiazolidinediones (TZDs)

Thiazolidinediones (TZDs) are potent agonists for peroxisome proliferator-activated receptor gamma (PPARγ) and exert strong stimulatory effects on fatty acid storage via adipogenesis and fatty acid flux into adipocytes in adipose tissue [51]. TZDs have shown the potential to enhance the brown features within WAT, leading to a reduction in obesity-related disorders [52,53]. In vivo or in vitro exposure of white adipocytes to TZDs activated the browning process, characterized by enhanced levels of mitochondrial biogenesis, oxygen consumption, and lipid oxidation [52,54]. Meanwhile, TZDs and other PPARγ ligands led to the suppression of multiple “bad” adipokines, such as resistin, α1-acid glycoprotein, and haptoglobin, which probably contributed to the anti-obesity effect of these drugs [55,56,57]. Unfortunately, TZDs have largely been withdrawn from the market due to their risks of inducing hepatotoxicity, myocardial infarction, bladder cancer, and heart failure [58]. Therefore, next-generation PPARγ agonists are warranted and are being tested for BAT and beige adipocyte activation.

#### 3.1.4. Others

Thanks to the development of high-throughput screening platforms, a broader scope of pharmacological reagents has been found with the potential for repurposing to treat obesity and metabolic dysfunction. A group of medicines has been demonstrated to activate UCP1 expression levels, including indirubin, rutin, and myricetin [47]. Indirubin is made from indigo naturalis and has a long history in traditional Chinese medicine. It has been mainly used for the prevention and treatment of cancers because of its suppressive effects on oncogenic gene expression [59]. It is now known that indirubin improves body weight, lipid accumulation, and glucose homeostasis in high-fat diet (HFD)-fed mice via enhancing thermogenesis in BAT and WAT [60]. Melatonin is recognized as a hormone that regulates sleep and circadian rhythms; it also shows a potential thermogenic effect. Oral melatonin-treated Zucker rats showed induced browning of inguinal WAT, with around double the expression level of UCP1 and PGC-1α [61]. Meanwhile, melatonin increased the inguinal temperature and made the rats more sensitive to acute cold exposure without physical activity modification [61]. The cellular screen approach is an alternative system with a directly visible fluorescence readout by which new candidates were identified. Sutent, for example, was found to be capable of upregulating UCP1 in BAT and elevating calorie consumption to protect against obesity [62].

In addition to unbiased screening-based studies, hypothesis-driven studies have also revealed some promising candidates. UCP1 is mechanistically regulated by signaling pathways such as Janus kinase (JAK) and PPARα. Tofacitinib and R604, two JAK inhibitors, induced brown-like metabolic properties, including elevated UCP1 expression and enhanced mitochondrial activity, in human adipocytes [63]. The activation of PPARα by fenofibrate, a PPARα agonist, triggered *UCP1* and other thermogenic gene expressions in BAT [64]. Along with insulin resistance, obesity and WAT dysfunction in obese mice were reversed [64]. Bexarotene, sold under the brand Targretin, is an antineoplastic agent used for the treatment of cutaneous T-cell lymphoma. A study showed that it enhanced the conversion of myoblastoma to brown adipocytes and promoted thermogenesis [65].

However, it should be noted that except for Myrbetriq, the current pro-browning evidence for the FDA-approved drugs mentioned earlier is based only on mouse or cell line studies. Clinical studies are needed to prove the effect of these drugs in enhancing energy expenditure and adipose tissue browning. In addition, repurposing raises concerns about the possible side effects of these drugs on obese/diabetic patients, posing a major obstacle. Table 1 summarizes the drugs examined in the studies described above.

### 3.2. Small-Molecule Compounds under Preclinical Trial Studies for Obesity and Metabolic Diseases

#### 3.2.1. Five Catalogs under Preclinical Trial Studies

As mentioned, PPARγ is a promising anti-obesity target, with multiple PPARγ agonists currently under development. Formononetin is a natural compound isolated from Astragalus membranaceus that shows putative PPARγ agonism activity. Formononetin protected mice from diet-induced obesity and facilitated a higher level of energy expenditure through increasing UCP1 following binding to PPARγ [66]. Mu Q et al. treated 3T3-L1 mature adipocytes with ginsenoside, the major active pharmacological component of ginseng. They found that 10 μM of ginsenoside Rb1 elevated basal and insulin-stimulated glucose uptake [67]. Moreover, ginsenoside Rb1 treatment also promoted adipocyte browning through the PPARγ signaling pathway, and its effect was abolished by the PPARγ antagonist [67]. Physical activity increases human energy expenditure and lowers the risk of obesity and T2DM [68]. β-aminoisobutyric acid (BAIBA) is a small-molecule myokine secreted from *PGC-1α*-expressing myocytes. In mice and humans, circulating BAIBA levels rose following exercise, ameliorating thermogenesis in WAT via PPAR [69].

Retinoid X receptor (RXR) is the heterodimeric partner for PPARγ. Both RXR and retinoid acid receptor (RAR) are endogenously activated by retinoid acid (RA). In 1995, Alvarez and Puigserver demonstrated that RA serves as a transcriptional coactivator contributing to UCP1 expression both in vitro and in vivo [70,71]. Fenretinide protected against weight gain and insulin resistance in obese mice [72]. Inhibition of preadipocyte differentiation was proposed as a possible mechanism [72]. However, fenretinide’s role as a thermogenesis inducer has not been directly tested yet. Benzoic acid and methoprene, working via RAR and RXR, respectively, significantly induced the mRNA expression of *UCP1* in BAT [73].

PR domain-containing 16 (PRDM16) is a 140 kDa zinc-finger PR (PRD1–BF1–RIZ1 homologous) domain-containing protein that was first identified by Nishikata et al. in 2003 [74]. Earlier studies found that PRDM16 was selectively expressed in brown adipocytes and had the capability to drive the brown phenotype and induce mitochondrial respiration [75]. It is also a coregulator of and interacts with PGC-1α/β or CtBPs to promote brown adipocyte-specific genes while suppressing white adipocyte gene expression [76]. Recently, rutaecarpine was identified as inducing the browning process in WAT after screening 500 natural compounds. With KEGG pathway analysis from RNA sequencing, it was proposed that the AMPK signaling pathway played a vital role as the downstream PRDM16 was activated [77]. Likewise, RGFP966, a selective class I histone deacetylase (HDAC3) inhibitor, was found to potentially drive the thermogenic pattern in brown and beige adipocytes in vitro, such as PGC-1α, UCP1, and FGF21 [78]. This impact of RGFP966 was proved to act through PRDM16 since PRDM16 knock-down blunted the effect. PRDM4 is another PRDM family member showing a positive function in stimulating browning in white adipose tissue. Butein is a biologically active flavonoid with an anti-cancer effect. It was identified as a potent means of inducing the expression of UCP1, increasing energy expenditure, and stimulating the generation of thermogenic adipocytes, thus highlighting a PRDM4-dependent pathway [79].

Bile acids (BAs) are natural products of the liver that participate in cholesterol catabolism and lipid absorption to reverse diet-induced obesity. Recent studies have shown that BAs are capable of enhancing energy expenditure in BAT and oxygen consumption in brown adipocytes [80]. The rising level of cAMP directly regulates these impacts after BAs bind to the G-protein-coupled receptor TGR5. Additionally, the genes involved in energy metabolism and uncoupling, iodothyronine deiodinase type 2 (*DIO2*), peroxisome proliferator-activated receptor γ coactivator-1α (*PGC-1α*), and *UCP1*, were found to be remarkably elevated with an increasing concentration of plasma BAs [81]. However, other hormonal functions of BAs via the farnesoid X receptor might have been overlooked, which makes them a less viable candidate.

During BA synthesis, liver X receptors (LXRs) are essential nuclear receptors with two isoforms (LXRα and LXRβ). New evidence has emerged that LXRs and PPARγ work either together to promote lipogenesis and differentiation in adipocytes or in an antagonistic manner to induce insulin resistance and suppress adiponectin signaling [82,83,84]. Moreover, LXR agonists, such as TO901317 and GW3965, inhibit adaptive thermogenesis via downregulating *DIO2* and *UCP1*, respectively [85,86]. On the contrary, as a natural compound derived from *Rheum palmatum* L., rhein evokes UCP1 expression by antagonizing LXRs in BAT, thus maintaining the energy balance in mice [87]. Further clinical investigations are necessary in light of its multi-target character [88].

#### 3.2.2. Other Chemicals Showing Efficacy in BAT, Beige Adipocytes, or Both

Several other compound molecules acting through distinct mechanisms have been observed to activate BAT in preclinical models and serve as candidates for the clinical treatment of obesity and associated diseases. We now summarize these molecules according to their reported target cells/organs.

WWL113 and capsiate are two molecules that mainly contribute to BAT activation. WWL113 inhibits the lipolysis enzyme of mouse carboxylesterase 3 (Ces3) and the human orthologue CES1 [89]. A recent study proved that CES1 activity was doubled in obese T2DM patients compared with lean individuals, and obese T2DM patients were noted to produce excessive fatty acids that were deposited in ectopic tissues [89]. WWL113 treatment resulted in a predominant augmentation in mRNA levels of *UCP1* and thermogenic-related genes, including *PGC-1α* and *DIO2*, in BAT both in vivo and in vitro via the PPARα signaling pathway [90]. WWL113 exhibited a more robust response in the presence of adrenergic stimulation in that it increased the energy expenditure of mice without affecting their locomotor activity, food intake, or heart rate [90]. These data support the potential of WWL113 in stimulating BAT activity. Similarly, capsiate, extracted from red peppers, is a modified nonpungent capsaicin analog. After treatment with 10 mg/kg body weight for 2 weeks, mice exhibited elevated UCP1 levels in their BAT and raised oxygen consumption. In another study, capsiate activated the sympathetic nervous system in mice by promoting adrenaline secretion. These results suggest that capsiate is a promising means to achieve BAT activation [91,92].

Other small molecules reportedly show selective effects on beige adipocyte induction, including WIN18446, linifanib, KY19334, and TG003. Aldehyde dehydrogenase 1 family member A1 (ALDH1a1) belongs to the aldehyde dehydrogenase family and is the second enzyme in the oxidative pathway of alcohol metabolism. WIN18446 is an ALDH1a1-specific inhibitor. It has been demonstrated to suppress weight gain and decrease the amount of visceral adipose tissue in obese mice [93]. Another independent study determined the possible underlying mechanism, indicating that ALDH1a1 ablation induced a BAT-remodeled transcriptional process in WAT, which enhanced mitochondrial respiration and adaptive browning via retinaldehyde and acetate [94,95]. In a study using a UCP1 reporter cell, linifanib was found to increase the expression of UCP1 via inhibiting STAT3 phosphorylation [96]. The Wnt/b-catenin signaling pathway is a negative regulator in adipogenic differentiation. In mice fed a high-fat diet, KY19334, a Wnt/b-catenin pathway activator via CXXC5–disheveled interaction, repressed adipogenesis and ameliorated insulin resistance and adipocyte hypertrophy, resulting in lower weight gain and potentiating browning [97]. PGC-1α is a substrate for Clk1 kinases, and inhibition of PGC-1α phosphorylation resulted in adipocyte beiging, indicating that Clk1 suppression may stimulate beige-like adipocytes. TG003, a Clk1 inhibitor, increased the number of mitochondria and induced fewer and smaller lipid droplets in adipocytes [98].

Considering the similar functions of brown and beige adipocytes, it is not surprising that several molecules show efficacy in activating both BAT and beige adipocytes. Nuclear factor kappa B (NF-κB) activation drives the activation of noncanonical IκB kinases IKKε and TANK-binding kinase 1 (TBK1) in adipose tissue and liver, subsequently counteracting energy storage [99]. It is intriguing that amlexanox, an inhibitor of these two kinases, increases thermogenesis and elevates energy expenditure; it has also been found to cause weight loss and ameliorate insulin resistance in mice with obesity [99]. Furthermore, the small-molecule compound BAY 41-8543 against soluble guanylyl cyclase (sGC) showed efficacy in protecting against diet-induced weight gain as a result of enhancing whole-body energy expenditure, enhancing the differentiation of brown adipocytes and inducing the thermogenesis of white adipocytes [100]. BIBO3304 is a selective antagonist of peripheral Y1R. It specifically induces thermogenesis in BAT and browning of WAT through the UCP1-dependent pathway [101]. BIBO3304 improves glucose homeostasis by driving Akt activity in BAT [101]. New data also provided the novel insight that a natural compound, harmine, is a thermogenic activator in both white and brown adipocytes, mediated by the RAC1/MEK/ERK pathway [102]. Studies have shown that berberine has a range of metabolic benefits, such as improving insulin resistance and hyperlipidemia. Zhang et al. found that in berberine-treated mice, fatty acids became the preferred fuel and generated more heat during cold exposure. Consistent with these processes, berberine also induced a thermogenic program in BAT and WAT via AMPK/PGC-1α [103]. However, the underlying mechanisms regarding the toxicity and side effects of these compounds in humans await further clarification.

Likewise, there is not enough current evidence to test these candidates on humans. The pharmacodynamics, pharmacokinetics, and toxicity of these compound candidates must be comprehensively studied before they can be further translated into clinical trials. Furthermore, although UCP1 was deemed the exclusive effector for thermoregulation, it is now clear that adipose thermogenesis is engaged by both UCP1-dependent and -independent mechanisms [17,18,104,105,106]. It is expected that new classes of pharmacological molecules targeting the non-canonical thermogenic pathways will be characterized and serve as novel drug leads for obesity and metabolic diseases. Table 2 summarizes the small-molecule compounds mentioned above.

## 4. BAT Transplantation to Counteract Metabolic Disorders

**Figure 2:**
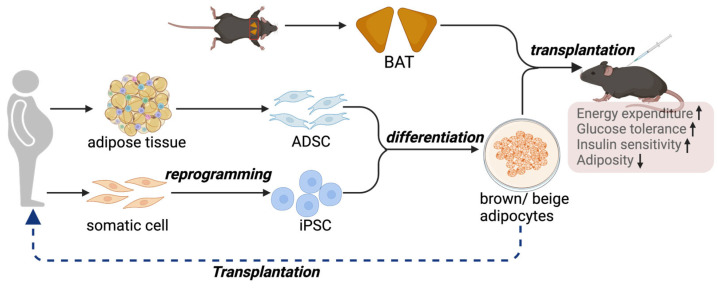
BAT, or thermogenic adipocyte transplantation, represents an alternative approach to counteract metabolic disorders. A proof-of-concept study demonstrates the therapeutic potential of thermogenic adipocytes and adipose tissue in protection against obesity and metabolic diseases. In the clinical setting, iPSCs or ADSCs are possible sources of precursors for brown and beige adipocytes.

Although activating brown and beige adipocytes is a feasible strategy for managing metabolic diseases, one of the major clinical issues is that adult humans have only a limited amount of BAT [107]. Additionally, BAT mass further declines in aging and diabetic conditions [108], making the pharmaceutical method less feasible in older individuals and diabetic patients. Furthermore, heterogeneity has been uncovered within BAT, where distinct subtypes of brown adipocytes with different thermogenic activities are present [24]. Therefore, transplantation of BAT to increase its mass and activity is emerging as an alternative approach to reduce the comorbidities associated with obesity (Figure 2).

### 4.1. BAT Transplantation: Proof-of-Concept Studies

The first attempt at BAT transplantation was carried out in 1960, predominantly aiming to delineate the in vivo function of BAT in mice [109]. Since then, a series of studies have been conducted to evaluate the metabolic outcomes of BAT transplantation. BAT was collected and transplanted into the subcutaneous region, visceral cavity, or dorsal inter-scapular region of HFD-induced obese mice [110,111,112]. In one of these studies, BAT was subcutaneously transplanted into the dorsal inter-scapular region of recipient mice, which then exhibited reduced weight gain, decreased liver mass, improved glucose uptake, raised insulin responsiveness, and increased body temperature [110]. BAT transplantation increased the level of circulating adiponectin, whereas it reduced the levels of circulating free T3 and T4, which regulate thyroid hormone sensitivity in peripheral tissues. Higher expression levels of fatty acid oxidation genes and improved glucose uptake in primary BAT were observed after BAT transplantation [113]. Gunawardana et al. investigated the effect of BAT on glucose metabolism in a mouse model with type 1 diabetes [112]. Embryonic BAT was obtained and transplanted subcutaneously into streptozotocin (STZ)-treated recipient mice. Mice with BAT transplantation exhibited normoglycemia, reduced tissue inflammation, improved glucose tolerance, and reversed clinical diabetic symptoms such as polyuria, polydipsia, and polyphagia [112]. Furthermore, elevated serum levels of leptin, adiponectin, and insulin-like growth factor 1 (IGF-1) were observed after BAT transplantation. However, it remained unaddressed whether the increased levels of these adipokines came directly from the transplanted BAT or via an indirect mechanism to improve the function of the endogenous adipose tissues. Since IGF-1 is one possible candidate for activating the insulin receptor, the authors proposed that the insulin receptor was activated, leading to an improvement in glucose homeostasis in their model. Indeed, the IGF-1 mechanism was further investigated in a non-obese diabetic recipient [114]. The success rate was elevated to 57% in adult BAT transplantation with IGF-1 supplementation [115]. It is also worth noting that BAT transplantation was accompanied by a significant increase in the expression of β3-AR in WAT and mitochondrial-specific OXPHOS proteins in endogenous BAT [116], suggesting that BAT transplantation increased whole-body thermogenesis and reduced obesity and related diseases by activating endogenous BAT.

### 4.2. Practicality and Ethical Concerns

Several concerns compromise the practicality of BAT transplantation from a human donor as a therapy. As in other organ donation cases, the high cost, availability of organ donors, and use of immunosuppressive medication are obstacles to be overcome before BAT transplantation can be deemed as a possible therapy. Furthermore, it should be noted that we currently lack unified standards for a “qualified BAT donor”. A comprehensive and standardized guideline to quantitively evaluate the amount and activity of functional and healthy BAT in adult humans across different sexes and ethnic groups is urgently needed. Furthermore, unlike other solid organ transplantations, the anatomical location of functional BAT in adult humans is heterogeneous and scattered throughout the body. In most cases, BAT is combined with WAT. Therefore, further elucidation is necessary to identify the location of the adipose tissue depot to be biopsied. Likewise, the location of transplantation to the BAT donor in humans is not yet optimized.

What is equally important is evaluating the short- and long-term health and psychological risks of post-surgical procedures. Answers to these questions are key to addressing the ethical considerations in BAT transplantation because BAT transplantation involves the removal of a healthy organ from the donor. Therefore, whether the traditional first rule of medicine—primum non nocere (above all, do no harm)—will be violated needs to be considered.

## 5. Brown and Beige Adipocyte Transplantations as Cell Therapies for Metabolic Diseases

Owing to the limitations of BAT transplantation, therapies using the in vitro culture, expansion, and differentiation of brown and beige progenitor cells to functional thermogenic adipocytes are emerging as alternative approaches and more feasible sources of cells for transplantation (Figure 2). The transplanted adipocytes can be derived from human adipose-derived mesenchymal stromal cells (ADSCs) or human induced pluripotent stem cells (hiPSCs).

### 5.1. ADSCs for Brown and Beige Adipocyte Differentiation and Transplantation

Brown adipocytes were induced from ADSCs and then transplanted into HFD-fed obese mice [117]. Very-low-density lipoprotein (VLDL) and low-density lipoprotein (LDL) levels declined in the circulation of obese mice after transplantation, accompanied by an increasing HDL level [117]. Additionally, the concentrations of proinflammatory cytokines, including IL-6, tumor necrosis factor-alpha (TNF-α), and IL-1β, were diminished by inhibition of the ITGAM/NF-κB-mediated proinflammatory responses and polarization of M2 macrophages. This study indicated that the ADSC-derived brown adipocytes could promote lipid catabolism and alternative polarization of M2 macrophages to ameliorate adipose inflammation in obese animal models. Similarly, a human ADSC suspension, conditioned medium, and cell lysate were intramuscularly injected into obese mice, resulting in a remarkable improvement in insulin resistance accompanied by a decrease in oxidized LDL and IL-6 [118]. In addition, mice receiving the human ADSC suspension injection exhibited lower lipid content and macrophage infiltration in the liver and adipose tissue, respectively; enhanced glucose tolerance; and pancreatic islet hypertrophy [118]. Recently, the generation of beige adipocytes from human ADSCs in a serum-free medium was reported [119]. The derived beige adipocytes showed a similar molecular profile to primary beige adipocytes isolated from human tissue and exhibited uncoupled mitochondrial respiration and cAMP-induced lipolytic activity. Transplantation of these beige adipocytes increased whole-body energy expenditure and oxygen consumption, and reduced body weight in recipient mice.

### 5.2. hiPSC-Derived Brown and Beige Adipocytes as Sources of Transplantation

hiPSCs were first generated in 2007 [120,121]. hiPSCs are generated from various somatic cells and self-renew in vitro; therefore, they represent a more accessible and unlimited source of brown and beige precursor cells. The differentiation ability of hiPSCs lays the foundation for cell therapies and has the potential to counteract obesity and associated comorbidities [120]. Significant advances have been made in the area of hiPSC-derived brown and beige adipocytes, showing promise for obesity treatment.

With the aim of treating patients with lipodystrophy, Taura et al. were the first to demonstrate the adipogenic potential of hiPSCs [122]. Approximately 15% of the induced cells were lipid-containing. Key adipogenic markers were observed in the hiPSC-derived adipocytes, including *CEBPα*, *PPARγ2*, and fatty-acid-binding protein 4 (*FABP4*). However, in this study, the phenotype of the generated adipocytes, i.e., whether they were white or brown, was not examined. In another study, hiPSC-derived fibroblasts were selected and induced into white adipocytes and brown adipocytes using the gene transfer method [123]. In this approach, the hiPSCs were driven into embryoid bodies, followed by generation into mesenchymal progenitor cells (MPCs). The MPCs were then programmed to express *PPARγ2* alone or combined with *CEBP-β* and/or *PRDM16*. After culturing in an adipogenic cocktail (insulin, 3-isobutyl-1-methylxanthine, dexamethasone) and doxycycline for 14–16 days and further maintenance without doxycycline until 21 days, these cells differentiated into white or brown adipocytes with a high efficiency of 85–90% [123]. These adipocytes exhibited the properties of mature adipocytes, such as expression of the mature adipogenic markers *FABP4*, *PPARγ2*, and *CEBPα*. They also showed adipocyte functions, including lipid metabolism and glucose uptake. Notably, after these mature adipocytes were engrafted into the recipient mice for 4–6 weeks, ectopic fat pads were observed with the morphological and functional properties of white and brown adipose tissue. However, no evidence has yet proven that these programmed cells expressing mature adipocyte markers can mimic physiological adipogenesis in vivo.

Studies have reported the successful differentiation of brown adipocytes without gene transfer [124,125,126]. Nishio and colleagues reported for the first time a highly efficient (>90%) differentiation method to induce hiPSCs into functional brown adipocytes via a specific hemopoietin cocktail without gene transfer [124]. On the other hand, hiPSCs were differentiated into white adipocytes without a hemopoietin cocktail [124]. The resultant brown adipocytes were functional since they exhibited potent thermogenic activation and mitochondrial respiratory response under β-adrenergic receptor exposure, accompanied by increased lipid and glucose tolerance. Moreover, the immune-competent mice showed metabolic improvements after hiPSC-derived brown adipocyte transplantation. Another method was described to achieve the direct differentiation of hiPSCs into brown and white adipocyte progenitors without gene transfer [125]. Treatment with the TGFβ pathway inhibitor SB431542 and ascorbic acid and EGF promoted hiPSCs to brown adipocyte differentiation, thus highlighting the critical role of the TGFβ pathway in switching off hiPSC-brown adipogenesis [125]. Most recently, hiPSCs were selectively induced into functional brown adipocytes via the paraxial mesoderm progenitor stage at high efficiency, characterized by increased rates of uncoupled respiration, glycolysis, and lipolysis [127]. Afterward, they were transplanted into the inter-scapular region of mice and showed thermogenic activity. That is, the recipient mice had enhanced respiratory exchange rates, metabolic activity, and whole-body energy consumption. Moreover, transplanted brown adipocytes decreased circulating glucose levels in STZ-induced diabetic non-obese diabetic/severe combined immunodeficiency (NOD/SCID) mice.

Efforts are also underway to generate beige adipocytes from hiPSCs. Guenantin et al. reported a straightforward and efficient procedure to derive functional beige adipocytes from hiPSCs through the mesodermal and adipogenic progenitor state [128]. These hiPSC-derived beige adipocytes expressed the beige-specific markers, displayed higher expression of thermogenic genes (but not *UCP1*), increased mitochondrial content, and improved oxygen consumption after cAMP analog stimulation. hiPSC-derived beige adipocytes formed a well-organized and vascularized adipocyte tissue with a higher glucose uptake rate after transplantation. In contrast, Aaron Brown’s team described the generation of human beige adipocytes from hiPSCs in a stepwise manner via forkhead box F1 (FOXF1+) splanchnic mesoderm, mural-like MSCs, and preadipocytes [129]. The mature hiPSC-derived beige adipocytes showed upregulated *UCP1* expression and uncoupled respiration. Cytokines were also secreted from hiPSC-derived beige adipocytes to improve insulin sensitivity and glucose uptake. At the molecular level, progenitors expressed beige/brite markers such as *CD137* and *TMEM26* but not the brown-specific marker *ZIC1*.

### 5.3. Practicality and Ethical Considerations for Brown and Beige Cell Therapies

Compared to BAT transplantation, the acquisition of brown and beige adipocytes in vitro is a more feasible means of transplantation. ADSCs can be harvested from the abdomen, thighs, flanks, and axilla using liposuction or direct excision techniques [130]. However, the in vitro expansion of ADSCs remains limited, making hiPSCs a superior and unlimited cellular source for brown and beige adipocyte differentiation, considering that hiPSCs can be derived from various skin, blood, and urine somatic cells [131,132,133,134]. However, knowledge about the developmental origins and pathways of human brown and beige adipocytes is still limited, especially regarding how the precursor cells of brown and beige adipocytes are developmentally derived. Consequently, compared to the in vitro differentiation from ADSCs, highly efficient differentiation protocols to generate authentic and functional thermogenic adipocytes from hiPSCs are still lacking and need to be developed.

Apart from the in vitro acquisition of functional brown and beige adipocytes, additional considerations and challenges remain. In particular, how long these cells stay alive/functional in the human body, especially in the “pathological” micro-environment of diabetic and aging patients, and how these transplanted cells react to endogenous stimuli and cause unforeseeable consequences require further investigation. The reactions between the physical body and differentiated cells determine how long the transplanted cells can stay alive and functional within the human body, thus determining the frequency of transplantation. If repeated transplantations are needed, additional financial considerations and safety issues must be considered. Whether the differentiated cells should be transplanted by local injection, intravenous infusion, or kept in macroencapsulation devices is another issue for discussion. In the case of allogeneic transplantation, the use of immunosuppressive medicine is critical to cell survival. Therefore, the appropriate immunosuppressive protocol requires further investigation before clinical use.

Meanwhile, using either ADSCs or hiPSCs involves ethical issues, and the donation should be governed by applicable laws and regulations to ensure the appropriate use of the cells. Table 3 summarizes all the cell transplantation therapies mentioned above.

## 6. Conclusions

The discovery of brown and beige adipocytes in human adults has inspired substantial efforts to develop new medicinal strategies targeting these thermogenic adipocytes. The original idea that these energy-dissipating adipocytes reverse the positive energy balance has been expanded in clinical and animal-based studies. Brown and beige fat has been directly implicated in glucose and lipid metabolism, the suppression of chronic inflammation, and hormone production [17,27,135,136]. Therefore, regimens to trigger the activity of brown and beige adipocytes are showing therapeutic potential beyond obesity and its related comorbidities, such as atherosclerosis, arterial hypertension, and polycystic ovary syndrome (PCOS). However, it should also be remembered that, currently, the downsides of these adipocytes are less clear and must be comprehensively evaluated. A balance between efficacy and safety must be reached. Hence, as illustrated above, brown and beige adipocyte-based therapies are full of promise, challenges, and pitfalls. A deeper understanding of their mechanisms of action, biological functions, and biogenesis is a prerequisite for eventually achieving effective and safe therapies for obesity, metabolic abnormalities, and a wider range of other diseases. Finally, in addition to the above scientific perspectives, safety and ethical issues should be considered and discussed carefully before brown and beige adipocytes can be applied clinically. Applicable laws and regulations governing related therapies will be warranted. 

## Figures and Tables

**Table 1:** FDA-approved drugs showing the potential to promote adipose browning.

Name	Original Purpose	Reference
MIRABEGRON	Overactive bladder	[11,39]
GLP-1RA	Type 2 diabetes	[47,48,49,50]
TZDs	Type 2 diabetes	[52,53,54]
INDIRUBIN	Chronic myeloid leukemia	[60]
MELATONIN	Insomnia	[61]
SUTENT	Gastrointestinal stromal tumors, renal cell carcinoma	[62]
TOFACITINIB	Rheumatoid arthritis, psoriatic arthritis, ankylosing spondylitis, juvenile idiopathic arthritis, ulcerative colitis	[63]
FENOFIBRATE	Hypertriglyceridemia	[64]
BEXAROTENE	Cutaneous T-cell lymphoma	[65]

**Table 2:** Small compounds showing browning potential.

Chemicals	Receptors/Downstream Target	Target	Models	Reference
FORMONONETIN	PPARγ	Beige adipocyte	3T3-L1	[66]
GINSENOSIDE	PPARα	Beige adipocyte	3T3-L1	[67]
BAIBA	PPARγ	BAT; beige adipocyte	Mouse; human	[69]
RETINOID ACID	RXR, RAR	BAT	Mouse	[70,71]
FENRETINIDE	RXR, RAR	Adipocyte differentiation and hypertrophy	3T3-L1	[72]
BENZOIC ACID	RAR	BAT	Mouse	[73]
METHOPRENE	RXR	BAT	Mouse	[73]
RUTAECARPINE	PRDM16	Beige adipocyte	Mouse	[77]
RGFP966	HDAC3	BAT; beige adipocyte	Mouse; human	[78]
BUTEIN	PPAR	Beige adipocyte	Mouse	[79]
BILE ACID	TGR5	BAT	Mouse; human	[80,81]
RHEIN	LXRs	BAT	Mouse	[87]
WWL113	CES1	BAT	Mouse; human	[90]
CAPSIATE	Sympathetic nervous system	BAT	Mouse	[91,92]
WIN18446	ALDH1a1	Beige adipocyte	Mouse	[93]
LINIFANIB	STAT3	Beige adipocyte	Mouse	[96]
KY19334	Wnt/b-catenin	Beige adipocyte	Mouse	[97]
TG003	Clk1	Beige adipocyte	3T3-L1	[98]
AMLEXANOX	TBK1	BAT; beige adipocyte	Mouse	[99]
BAY 41-8543	sGC	BAT; beige adipocyte	Mouse; human	[100]
BIBO3304	Y1R	BAT; beige adipocyte	Mouse; human	[101]
HARMINE	(DNA binding protein 4) CHD4	BAT; beige adipocyte	Mouse	[102]
BERBERINE	ERK/p38 MAPK	BAT; beige adipocyte	Human	[103]

**Table 3:** Cell transplantation therapy for improvement of metabolism.

Source	Recipient	Effects	Reference
MICE BAT	HFD-fed obese mice	Reduced weight gain; Decreased liver mass; Improved glucose uptake and insulin responsiveness; Increased body temperature.	[110]
MICE EMBRYONIC BAT	Type 1 diabetes mice	Normoglycemia; Reduced tissue inflammation; Improved glucose tolerance; Reversed clinical diabetic symptoms.	[112]
MICE BAT	Ob/Ob mice BAT-deficient obese mice	Increased circulating adiponectin; Improved glucose uptake.	[113]
MICE BAT	Hyperglycemic non-obese diabetic mice	Elevated serum level of IGF-1.	[115]
MICE BAT	Ob/Ob mice	Increased β3-AR expression in WAT; Activated endogenous BAT.	[116]
MICE ADSC-DERIVED BROWN ADIPOCYTES	HFD-fed obese mouse	Decreased levels of VLDL and LDL; Increased level of HDL.	[117]
HUMAN ADSCs	HFD-fed obese mouse	Improvement in insulin resistance; Decreased oxidized LDL and IL-6.	[118]
HUMAN ADSC-DERIVED BEIGE ADIPOCYTES	NOD/SCID mice	Increased whole-body energy expenditure and oxygen consumption; Reduced body weight.	[119]
HIPSC-DERIVED BROWN ADIPOCYTES	Immune-competent mouse	Metabolic improvements.	[125]
HIPSC-DERIVED BROWN ADIPOCYTES	Mouse	Enhanced respiratory exchange rates, metabolic activity, and whole-body energy consumption.	[127]
HIPSC-DERIVED BROWN ADIPOCYTES	NOD/SCID mouse	Decreased circulating glucose levels.	[127]
HIPSC-DERIVED BEIGE ADIPOCYTES	FoxN1^Nu^ athymic mice	Higher glucose uptake rate.	[128]
